# Physical Activity: A Missing Link in Asthma Care

**DOI:** 10.3390/jcm9030706

**Published:** 2020-03-05

**Authors:** Marios Panagiotou, Nikolaos G. Koulouris, Nikoletta Rovina

**Affiliations:** 1st Deptartment of Respiratory Medicine, National and Kapodistrian University of Athens, Sotiria Thoracic Diseases General Hospital, 115 27 Athens, Greece; koulnik@med.uoa.gr (N.G.K.); nikrovina@med.uoa.gr (N.R.)

**Keywords:** asthma, lung function, daily life physical activity, exercise, activity monitoring, accelerometer

## Abstract

Asthma is the commonest respiratory disease and one of unceasingly increasing prevalence and burden. As such, asthma has attracted a major share or scientific interest and clinical attention. With the various clinical and pathophysiological aspects of asthma having been extensively investigated, the important association between asthma and physical activity remains underappreciated and insufficiently explored. Asthma impacts adversely on physical activity. Likewise, poor physical activity may lead to worse asthma outcomes. This concise clinical review presents the current recommendations for physical activity, discusses the available evidence on physical activity in asthma, and examines the causes of low physical activity in adult asthmatic patients. It also reviews the effect of daily physical activity and exercise training on the pathology and clinical outcomes of asthma. Finally, it summarizes the evidence on interventions targeting physical activity in asthma.

## 1. Introduction

Asthma is a heterogeneous chronic inflammatory disease of the airways that is defined by the history of respiratory symptoms such as wheeze, shortness of breath, chest tightness and cough that vary over time and in intensity, together with variable expiratory airflow limitation [1]. In the present era, asthma is estimated to affect as many as 339 million people across all ages worldwide, carries a substantial burden of morbidity and mortality and constitutes a major source of global economic burden in terms of both direct and indirect costs [2].

Physical activity often provokes asthma-related symptoms reflecting the nature or insufficient control of asthma. Thus, asthmatic patients may often intuitively or purposely avoid exercise and adopt a sedentary lifestyle. Not long ago, asthmatic patients were in fact regarded as chronically infirm individuals who needed to be sheltered and avoid physical exertion in order to prevent severe asthma attacks [3]. However, advances in understanding of the pathobiology and the management of the disease, including the development of a wide range of potent drugs and effective delivery devices, avoidance of triggering factors, patient education, and asthma action plans, have induced a paradigm shift in the perception of asthmatic patients and therapeutic goals. In the modern era, the ultimate goals of asthma management include the minimization of symptoms so that patients can maintain normal levels of activity and achieve good quality of life [1,3]. Accordingly, the Global Initiative for Asthma (GINA) recommends people with asthma to engage in regular physical activity in order to improve their general health [1].

Increasing physical activity may actually be an important overlooked link in optimising the management of asthma, especially the severe type of asthma and specific asthmatic populations. Accumulating evidence links enchanced physical activity with favourable outcomes, including better overall asthma control, less exacerbations, and lower healthcare use [4]. Therefore, physical activity in asthma warrants attention and focus. This concise clinical review presents the current recommendations for physical activity, discusses the available evidence on physical activity in asthma, and examines the causes of low physical activity in adult asthmatic patients. It also reviews the effect of daily physical activity and exercise training on the pathology and clinical outcomes of asthma. Finally, it summarizes the evidence on interventions targeting physical activity in asthma.

## 2. Physical Activity Nomenclature and Recommendations

Physical activity is comprised of bodily movements produced by the skeletal muscles that result in an increased metabolic rate over that of resting energy expenditure [5]. Physical activity in daily life (PADL) encompasses household, occupational, transportation, conditioning, athletics, sports, or other activities in the context of daily, family, and community activities, and can be described by dimensions of intensity, frequency, duration, mode, and context. As such, PADL satisfies the core requirement of a meaningful clinical trial endpoint defined to be a direct measure of how a patient feels, functions or survives where function refers to the ability to carry out normal daily activities [6]. Accordingly, PADL has been shown to be an important dimension of health-related quality of life in both health [7,8] and disease [9,10] and is becoming of increasing research and clinical interest.

For the purpose of improving health outcomes, the mode, intensity, frequency and duration of physical activity must be collectively considered [11]. In order to improve cardiorespiratory and muscular fitness, bone health, reduce the risk of noncommunicable disease and depression, the World Health Organization recommends that adults should engage in at least 150 min of moderate-intensity aerobic physical activity (defined as activity requiring 3–6 metabolic equivalents; METs) throughout the week or do at least 75 min of vigorous-intensity aerobic physical activity (requiring > 6 METs) throughout the week or an equivalent combination of moderate-and vigorous-intensity activity (MVPA), all performed in bouts of at least 10 min duration [12,13]. These refer to the minimal amount of activity recommended to achieve substantial benefits over and above the routine light-intensity activities of daily living. For additional health benefits, older adults should increase their moderate intensity aerobic physical activity to 300 min per week or engage in 150 min of vigorous-intensity aerobic physical activity per week, or an equivalent MVPA. These recommendations are relevant to all healthy adults aged 18 and above, irrespectively of gender, race, ethnicity or income level. They also apply to individuals in this age range with chronic noncommunicable conditions not related to mobility [12,13], including asthma. Older adults, 65 years and above, with poor mobility, are also advised to perform physical activity to enhance balance and prevent falls on three or more days per week and muscle-strengthening activities, involving major muscle groups on two or more days a week; when older adults cannot do the recommended amounts of physical activity due to health conditions, they should be as physically active as their abilities and conditions allow [13]. Expressed in metabolic equivalents-minutes (MET-min), the minimal recommended amount of MVPA lies in the range of 450 to 750 MET-min per week [14]. Translated to step count, the daily recommended amount of MVPA, accumulated in addition to habitual daily activities (taken in the course of free-living and not necessarily of at least moderate intensity), corresponds to ≥ 10,000 steps per day in healthy adults [15] and approximately to 7000–10,000 steps/day in healthy older adults [16].

Physical activity may not be used interchangeably with exercise. Exercise is a subcategory of physical activity that is planned, structured, and repetitive and has as a final or an intermediate objective the improvement or maintenance of one or more components of physical fitness [5]. Physical activity may also not be confused for maximal exercise capacity. The latter can be defined as the maximum amount of physical workload that an individual can sustain through a coordinated cardiovascular, respiratory, and neural response along with the action of exercising muscles. An accurate assessment of exercise capacity requires that maximal physical exertion is sufficiently prolonged to reach a stable (or steady state) effect on the circulation and that the pattern of response is consistent when exertion is repeated, and it is best conducted by cardiopulmonary exercise testing [17]. However, maximal exercise performance assessed by standardized exercise protocols does not correlate sufficiently with daily life activities in patient populations [18,19]. Finally, functional exercise capacity as assessed by walking tests also does not identify with PADL. The self-paced 6-min walk test assesses the submaximal level of functional capacity and, because most activities of daily living are performed at submaximal levels of exertion, the 6-min walk distance has been though to better reflect the functional exercise level for PADL [20]. However, various reports do not confirm a sufficient correlation between PADL and functional exercise capacity [21,22].

Physical activity is best evaluated objectively by accelerometers. Accelerometers are wearable monitor devices that measure multiaxial accelerations of the body segment to which they are attached. The signal is usually filtered and pre-processed by the monitor to obtain activity counts, i.e., accelerations due to body movement. The amount of sedentary and physical activity time and the intensity of activity may be obtained by classifying activity counts accumulated in a specific time interval (epoch length) using a set of cut-points, i.e., intensity thresholds for classification of the intensity of physical activity [23]. The energy expenditure during physical activity or sleep-related measures may also be estimated by applying algorithms to objectively-determined activity counts, whereas new methods to estimate these variables from raw acceleration signals (gravity units) instead of activity counts have also been developed [23]. Accelerometers offer a valid, objective alternative to self-report questionnaires, which suffer from recall bias and insufficient validity [24], although their use in population-based or longitudinal studies may be less feasible.

## 3. Physical Activity in Asthma

Population-based evidence suggests that individuals with asthma have reduced capacity to undertake regular activities and work [25]. Earlier population-based evidence collectively suggests that individuals with asthma are less likely to engage in physical activity and less likely to engage in more intense exercise than those without asthma as well as less likely to engage in physical activity at recommended levels [26,27,28,29]. A recent systematic review among all available studies using a control group, identified 11 studies (asthma sample = 32,606) reporting less physical activity in asthma, and 6 studies (asthma sample = 7824) reporting no difference thus leading to the conclusion that people with asthma engage in less activity compared to controls [30]. Data on sedentary time in asthma are still scarce [30]. In two cross-sectional studies (asthma sample = 287) where sedentary time was examined using activity monitors, engagement in sedentary behavior was similar between asthmatics and controls but both groups were highly sedentary [31,32].

A systematic review of 4 studies using activity monitors concluded that people with asthma tended to accumulate less physical activity in terms of volume and/or intensity, than controls [30]. A meta-analysis of 7 studies (asthma sample = 526) reporting on accelerometer-counted daily steps revealed a mean of 8390 daily steps (95% confidence interval (CI) 7361–9419) among steps patients with mild, moderate or severe asthma [30]. The latter suggests that asthmatics fall into the “somewhat active” category of a step-defined ladder of physical activity [33] and most fail to meet current recommendation for physical activity [15]. However, this estimate may even be overoptimistic for specific subgroups in the asthma population which are both underrepresented and at risk for lower physical activity, such as those with severe asthma or obesity and the elderly.

Few cross-sectional studies have focused on the objective assessment of physical activity in the important population of patients with severe asthma. Severe asthma is defined as asthma that remains uncontrolled despite adherence with maximal optimized therapy and treatment of contributory factors or that worsens when high dose treatment is decreased [1,34]. Severe asthma has an estimated prevalence of 5–10% in the total asthma population, and is important because it impacts considerably on morbidity and mortality and accounts for a major share of healthcare utilization and cost as well as socioeconomic cost in asthma [34]. Patients with severe asthma suffer a heavy burden of symptoms, exacerbations and medication side-effects that interfere with everyday living, sleeping and physical activity [1]. Unsurprisingly, evidence shows the level of PADL to decrease with loss of asthma control [32] and increasing asthma severity [35]. Studies in severe asthma are consistent in that patients with severe asthma present with considerably low levels of physical activity. Cordova-Rivera and colleagues [31] reported as low as 5362 (median 3999–7817) steps per day and 21.9 (12.8–37.9) min of MVPA per day in 61 patients with severe asthma. Compared with controls, individuals with severe asthma accumulated 31.4% (2,232) less steps per day and engaged in 47.5% less minutes per day in MVPA (*p* < 0.001 for both). Bahmer and colleagues [36] reported 6174 (4822–9277) daily steps and 125 (68–172) minutes of at least moderate activity per day in 63 patients with severe asthma, both of which were significantly lower compared to 83 patients with mild-to-moderate asthma (by 21% and 17%, respectively) and 29 healthy controls (by 31% and 23%, respectively), after adjusting for confounders. Of note, PADL in severe asthmatics was associated with impulse oscillometric airway resistance and small airway dysfunction, albeit not with gold-standard airflow limitation measured by forced expiratory volume in 1 s (FEV_1_) and peak expiratory flow rate (PEFR) [36]. This is important because peripheral airway involvement and functional status are highly relevant to the pathophysiology and clinical manifestations of asthma [37]. Hennegrave et al. [38] reported on a low daily step count of 6560 ± 3915 in a cohort of 23 patients with severe asthma. In line, we have previously reported findings on limited daily moving time (walking, stair climbing, and cycling; 88 ± 34 min) daily step count (7142 ± 2952), and weekly MET-min (509 ± 189) in 12 patients with severe asthma [39]; movement intensity measured in acceleration units was found to be 1.81 ± 0.18 m/s^2^, which compares with that of older patients with moderate-to-severe chronic obstructive pulmonary disease (COPD) [40].

Based on daily step count, these findings place patients with severe asthma more into a “low-active” population [33].

However, PADL has not been unequivocally associated with the severity of asthma. In children [41] and adolescent [42] patients with asthma, physical activity levels were shown to be independent on asthma severity. In the study by Hennegrave et al. [38], adult patients with severe asthma did not differ significantly from matched patients with mild/moderate asthma in terms of daily steps (6560 ± 3915 vs. 8546 ± 3431), time spent in MVPA (120 ± 54 vs. 121 ± 32 min/day), energy expenditure (EE) in MVPA (620 ± 360 vs. 660 ± 140 kcal/day), or total EE (2606 ± 570 vs. 2666 ± 551 kcal/day) (*p* ≥ 0.80 for all) and PADL was also not influenced by asthma control or per se. However, the impairment in FEV_1_, a representative marker of the magnitude of bronchial obstruction in asthma, was associated with the number of steps per day [38]. It is possible that the underpowered nature of the study contributed to some of these findings.

## 4. Causes for Low Physical Activity in Asthma

Compared to healthy controls, young asthmatic patients were found to have similar maximum heart rate but reduced maximal oxygen consumption (VO_2max_), anaerobic threshold and oxygen pulse, suggesting suboptimal fitness [43]. However, reduced fitness in asthmatic patients cannot not be attributed directly to airflow obstruction. Lung function (FEV_1_) in asthmatics did not correlate with VO_2max_, anaerobic threshold, or oxygen pulse either before or after bronchodilator, and asthmatic patients had sufficient ventilatory reserve to allow toleration of exercise workload adequate to enhance cardiovascular fitness [43]. Accordingly, nebulised bronchodilator therapy with salbutamol before exercise, albeit convincingly improved FEV_1_, had little effect on the cardiorespiratory responses such as maximal workload, oxygen uptake, heart rate, and ventilation to progressive maximal exercise in patients with mild asthma [44]. Also, although salbutamol administration increased tidal volume at maximal exercise, it did not change in perception of exertional breathlessness in asthmatic subjects [44]. Finally, the state of airway function—whether bronchodilated or bronchoconstricted—prior to exercise was not found to affect ventilation or operating lung volumes during exercise in mild asthmatics with normal maximal exercise capacity [45]. Therefore, evidence suggests a robust pulmonary system, at least in the mild asthmatics; one that is capable of adequately responding to the demand for acute increase in airflow necessitated by high-intensity aerobic exercise. Put otherwise, many patients with asthma become less fit and therefore, less likely to become physically active and achieve their maximum potential in lifestyle pursuits, than their pulmonary condition would allow [46].

Physical activity is a potent stimulus for asthma symptoms and vigorous physical activity is particularly associated with more asthma symptoms [47,48,49]. Emerging or worsening asthma symptoms during exercise may result in reduced exercise tolerance and fear of experiencing such symptoms may lead many people with asthma to intuitively, or pre-emptively avoid physical activity. Generally, the status of asthma control is an important determinant of physical activity in asthmatic patients [32]. In national representative studies in the United Kingdom [50] and the United States [51], patients with uncontrolled moderate-to-severe asthma were at higher risk for limitations in overall physical activity, outdoors activity and daily activity compared with patients with controlled asthma. Individuals with asthma also present paradoxical responses to physical activity. Up to 90% of asthmatic patients develop exercise-induced bronchoconstriction (EIB), a distinct form of bronchial hyperresponsiveness (BHR) defined as acute, transient narrowing of the airways during or immediately after cessation of exercise, or strenuous physical activity [52]. EIB in asthma reflects disease control and is thought to result from changes in airway physiology triggered by the large volume of relatively cool and dry inhaled air. Typical symptoms include dyspnoea, chest tightness, wheezing, or cough and may result in exercise avoidance. EIB may also occur in subjects without clinical asthma, particularly in children, athletes, individuals with atopy or rhinitis and after respiratory infections; earlier reports suggest a prevalence of 5% to 20% in the general population. EIB can more commonly diagnosed by a ≥10 % sustained decrease in FEV_1_ after a standardized exercise challenge test; other direct or indirect bronchoprovocative tests may also be used as surrogate diagnostic tools [52]. Finally, some atypical causes of exertional dyspnoea are more common in asthma, including psychogenic hyperventilation [53] and dysfunctional breathing [54]. Vocal cord dysfunction is also common in asthmatic patients [55] and recognized to be present in up to half of patients with severe asthma [56].

Demographic factors may also account for low activity in asthmatic patients. The impact of obesity and the aging on physical activity [56] should be considered for the large populations of obese [57] and elderly [58] people living with asthma, respectively. Evidence shows that obese patients with asthma present with low-level PADL and they most commonly fail to meet the international recommendations for physical activity [59]. Also, the decrease in activity tends to be more pronounced, or even exclusive, in older people with asthma than their younger counterparts [30]. Plausible biological decline in lung and systemic function, chronicity of asthma and airway inflammation, chronic smoking and accumulating comorbidities (see below) as well as previous beliefs on physical activity and asthma, may all synergistically render older asthmatics less active. Finally, physical activity in asthma seems to be influenced by sex as several studies reported lower physical activity in women with asthma compared to men [30]. This may reflect societal factors and seems to follow the trend in the general population.

Frequent asthma comorbidities including allergic dermatitis, chronic rhinosinusitis, arthritis, gastroesophageal reflux disease, hypercholesterolemia, and depression have also been associated with activity limitation in large-scale studies [51], and co-diagnosis of COPD, coronary artery disease, diabetes and depression was associated with asthma-related hospitalizations and emergency department visits in asthmatics older than 65 years [60]. Also, psychological distress, anxiety or depression, and decreased feelings of control, which are common in asthma patients, are significantly associated with physical health status [61] and physical activity [56]. Psychosocial factors are in fact thought to affect the pathogenesis and pathophysiology of asthma, either directly through autonomic, endocrine, immunological, and central nervous system mechanisms, or indirectly through personal motivations, self-conceptions, lifestyle aspects, health behaviours, and illness cognition, perception, and response including adherence to medication and avoidance of triggers [62]. Finally, restriction of physical activity leads to deconditioning and the establishment of a vicious circle of inactivity, deconditioning, and symptoms, which has also been implicated in low levels of physical activity among adults with asthma [4,63].

## 5. The Effect of Physical Activity in Asthma

The possibility of an etiological relation between physical activity and development of incident asthma has been investigated by several studies but the overall quality of evidence remains low due to cross-sectional design of most of the studies, self-reported diagnosis of asthma and measures of physical activity. More appropriate for the investigation of causal relationships are longitudinal studies in which the exposure (physical activity) precedes the outcome (onset of asthma). A systematic literature review of 5 such longitudinal studies (duration between 5 and 10 years, *n* = 85,117) indicated that physical activity is a possible protective factor against development of asthma (odds ratio for incident asthma 0.88 (95% CI 0.77 to 1.01) [64]. However, this meta-analysis was unable to control for potential confounding factors such as demographic and socioeconomic characteristics, obesity and smoking [64]. Examined separately, the longitudinal studies that tested for confounding factors, either do not provide strong evidence or do not actually confirm the association between physical activity and incident asthma [65,66,67]. In accordance, a subsequent study of 18,894 adults which controlled for age, sex, body mass index, smoking status, education, family history of asthma, social benefit, economic difficulties, and allergic rhinitis did not observe a decreased risk of incident asthma in physically active adults compared with inactive adults over an 11-year follow-up [68]. Finally, a prospective study that also checked for confounding factors, did not find an association between accelerometry-measured physical activity and development of wheezing or shortness of breath in pre-school children [69]. In contrast, in a single longitudinal study, there was a weak association between reduced physical fitness in childhood and the development of asthma during adolescence, whereas high physical fitness seemed to be associated with a lower risk for asthma development [70].

Population-based evidence has shown higher level of regular physical activity to reduce the risk of asthma exacerbations, independent of asthma severity and other covariates [71] and to be associated with lower health care use (physician consultations and overnight hospital stay) by asthmatic individuals compared to controls [72]. In a recent systematic review, higher levels of physical activity were collectively associated with better measures of lung function, asthma control, health status, exacerbations and health care utilisation [30]. Likewise, cross-sectional studies found sedentarism to be associated poorer lung function, asthma control and exercise capacity [31] and higher health care use [73]. Certainly, evidence on the dynamic interrelation between asthma and physical activity that derives from cross-sectional studies has limitations as it is inherently not suited for the conclusion of causal relations (besides the hypothesis that subjects with higher physical activity levels have a favourable effect on asthma outcomes, reverse causality is also possible). However, these findings should collectively encourage further research and the engagement of asthmatic individuals to regular physical activity. Increasing PADL via low-cost, accessible means (e.g., walking) may be a beneficial intervention for asthma, in addition to its known cardiometabolic and other health benefits [4]. Also, more research is warranted to explore the extent of sedentary behaviour in asthma and its association with adverse clinical outcomes. Promoting frequent and longer breaks of sedentary behaviour may even be a more achievable goal than increasing activity in people with asthma [30].

With regards to lung function specifically, three longitudinal studies provide the strongest evidence for a beneficial effect of physical activity on lung function in asthma. An observational population-based cohort study of 6790 people in Denmark with 153 having asthma at baseline, found that moderate to high physical activity was associated with less lung function decline among smokers, and the association was stronger in the subgroup with asthma than those without asthma, during the 3.7-year follow up [74]. In the asthmatic individuals, moderate to high physical activity ameliorated lung function decline by gaining 10 mL/year of FEV_1_ and 7 mL/year of forced vital capacity (FVC), as compared with the low physical activity group, whereas the corresponding figures in the non-asthmatics were 1 mL/year and 2 mL/year, respectively. [74]. In another population-based cohort of 1329 adults with asthma in Norway, there was some evidence of slightly less lung function decline in physically active participants compared with the inactive ones. On average, active asthmatics had 1.5–2.1% less decline in the FEV_1_/FVC ratio and 44–88 mL less decline in PEFR during the 11.6-year follow-up [75]. Finally, in a cohort of 201 individuals in Finland who were followed for 12 years after diagnosis of asthma, those with high physical activity had slower annual FEV_1_ (−41.4 mL vs. −58.8 mL, *p* < 0.001) and FVC (−29.3 mL vs. −43.6 mL, *p* < 0.018) decline compared with those with low physical activity [76]. Also, the high physical activity group had higher FEV_1_ values at follow-up, and higher FEV_1_/FVC ratios at diagnosis and follow-up [76]. Weak but significant associations between physical activity and lung function have also been reported by several cross-sectional studies, summarised elsewhere [30].

## 6. The Effect of Exercise Training in Asthma

To date, 10 randomised controlled trials (RCTs) have collectively reported benefits of supervised aerobic exercise training on a range of outcomes in adult patients with asthma such as exercise capacity, airway inflammation, disease exacerbation, clinical control, health-care use, psychosocial symptoms and asthma-related quality of life [30,77,78,79,80,81,82,83,84,85,86]. In the largest and most recent RTC including 89 subjects with mild or moderate asthma, aerobic exercise at least three times a week for ≥ 30 minutes plus muscle training, and stretching, improved asthma control measured by the Asthma Control Test questionnaire and reduced shortness of breath [78]. The effect of intervention on improving asthma control was 23% (risk reduction (RD) = 0.23, 95% CI 0.027–0.438; *p* = 0.0320) and 30.1% for shortness of breath by (RD = 0.301, 95% CI 0.109–0.492; *p* = 0.003). A meta-analysis of 17 RCTs, including 599 children and adult asthmatics, also reported that exercise training led to a significant improvement in days without asthma symptoms (mean difference (MD) 8.90 symptom-free days, 95 % CI 8.18–9.61, *p* < 0.001) [87]. These findings are promising although they concern relatively small groups of patients attending structured training sessions at well-resourced, designated centres or led by physiotherapists, which are not generalizable to many real-world settings [4]. The variability in exercise protocols among the studies (including various types of land-based exercise methods including walking, running, jogging, cycling, strength training, or a combination of these at both low and high-altitude settings, for variable duration between 3 and 24 weeks) reflects the knowledge gap on the optimal exercise regime and the absence of relevant guidelines.

From a physiological scope, exercise training evidently improves cardiopulmonary fitness, whereas its impact on lung function is less clear. A 2013 Cochrane metanalysis of RTCs of people over eight years of age with asthma showed significant improvement in VO_2max_ with land-based exercise training (MD 4.92 mL/kg/min; 95% CI 3.98–5.87; *p* < 0.00001; eight studies on 267 participants) without significant effects in other measures of pulmonary function such as FEV_1_, FVC, minute ventilation at maximal exercise or PEFR [11]. The improvement in cardiopulmonary fitness is important because it may reduce the risk of dyspnoea due to deconditioning and other conditions unrelated to airflow limitation that may mistakenly attributed to asthma [11]. This essentially enables larger breathing reserves for activities of daily life with a better effort-benefit ratio [78]. Also, there was some evidence to suggest that physical training may have positive effects on asthma symptoms and health-related quality of life, with the majority of studies producing a statistically and clinically significant benefit [11,88]. Another meta-analysis in children and adult asthmatics, also showed that exercise training led to a significant improvement in exercise capacity (MD 4.06 mL/min/kg, 95 % CI 3.02–5.10, *p* < 0.001 for VO_2max_; MD 24.03 W, 95 % CI 20.15–27.91, *p* < 0.001 for maximal working capacity; standardised MD 0.81, 95 % CI 0.13–1.48, *p* < 0.02 for exercise endurance) [87] but a questionable change in FEV_1_ (MD 0.09 L, 95 % CI 0.00–0.17, *p* = 0.05) compared with usual care. However, the analysis of relative within-group changes after exercise training (in both RCTs and controlled trials) showed small improvements in FEV_1_ (3 ± 7 %, *p* = 0.019) compared with control conditions and multiple linear regression modelling revealed that changes lung function, along with changes in BHR, contributed significantly to the observed improvement in the quality of life [87]. Nonetheless, the magnitude of the effect of exercise training on lung function may have been hampered by the limited time frame of these interventions—a hypothesis which is supported by the aforementioned evidence on the beneficial effect of enchanced PADL on lung function in asthma.

Swimming is often recommended as a form of exercise for asthmatics due to the humidified and warm air, low pollen count exposure and hydrostatic pressure on the thoracic wall [89], although some concerns exist about chlorine exposure with indoor pools [1]. In a systematic review of eight studies (*n* = 262) of children and adolescents with asthma, swimming programs were shown to increase cardiopulmonary fitness (mean increase in VO_2max_ 9.67 mL/kg/min, 95% CI 5.84–13.51 (2 studies; *n* = 32)) and improve lung function (FEV_1_; 0.1 L higher, 95% CI 0–0.2 (four studies; *n* = 113)) in a clinically meaningful fashion compared to usual care. [89]. However, there was no statistically significant difference between swimming and usual care/other physical activities on quality of life, asthma control, exacerbations and medication use to recommend swimming over other forms of physical activity [89]. Water-based exercises in adults have been less researched. A Cochrane systematic review of three available studies including 136 adult participants who followed swimming and water aerobics was unable to assess the place of water-based exercise in asthma due to the small number of participants, the clinical and methodological heterogeneity observed, and the high risk of bias assessed [90]. Nonetheless, engagement of patients with mild persistent asthma in recreational swimming in non-chlorinated pools, combined with regular medical treatment and education, led to better improvement in lung function and more significant decrease in BHR (2.01 vs. 1.75; *p* < 0.001) compared with standard care [91].

Changes in BHR with physical activity have also been reported following land-based exercise training. In a RCT by Franca-Pinto, aerobic training led to a reduction in BHR by 1 doubling dose (95% CI 0.3 to 1.7 doubling dose) in patients with moderate or severe asthma [84]. In that study, patients with higher airway inflammation (baseline exhaled nitric oxide levels (FeNO) ≥ 26 ppb and ≥ 3% sputum eosinophils) and worse asthma control also experienced reduction in sputum eosinophils and FeNO [84]. In an analysis of relative changes in available non-controlled, controlled and randomized controlled trials in children, adolescent and adult patients with asthma, exercise training in the form of land-based exercise or swimming was shown to improve BHR by 53% and EIB by 9% compared with control conditions; also, improvements in BHR explained part of the observed improvement in quality of life and exercise capacity [87]. Animal studies suggest that aerobic exercise training attenuates BHR via a mechanism that involves β2-adrenergic receptors [92].

Athletes with asthma represent a distinct exercising asthmatic population from which lessons can be gleaned. EIB (often without respiratory symptoms) and asthma occur frequently and increasingly in athletes, reaching an estimated prevalence between 30% and 70% among elite athletes, depending on the type of sport performed [93,94]. Athletes may have already had BHR and asthma before taking up sports, or may have developed them since becoming active in sports. Phenotypic distinction of asthma in athletes to “atopic asthma” defined by allergic sensitization, rhinitis, allergic comorbidities and increased FeNO; and “sports asthma”, defined by exercise-induced respiratory symptoms and BHR unrelated to allergy but related to specific type of sport and environment, has been proposed [95]. The development of BHR and asthma in athletes is possibly attributed to the high frequency of repeated physical strain and excessive ventilation occurring during training and competitions as well as the overexposure to environmental factors, allergens and irritants such as cold and dry air, air pollution and organic chlorine compounds. Thus, environmental setting and exposure during exercise training is an important determinant of development of and control of asthma. Accordingly, BHR, EIB and asthma are more common in endurance sports, particularly winter sports and swimming [93,96,97]. Nonetheless, as the various achievement of asthmatic athletes in competitive sports can attest, asthma can usually be well managed in athletes, most often with the use of maintenance inhaled glucocorticoids and inhaled short-acting β_2_-agonists along with warm-up before exercise and, therefore, allow for maximal performance. Of note, currently used inhaled β_2_-agonists have no performance-enhancing effect in athletes with or without asthma and are therefore permitted for use by the World Anti-Doping Agency (WADA; for salbutamol the maximum daily dose permitted being 1600 μg). However, their regular or frequent uses may lead to tolerance and decrease their bronchoprotective effect during exercise [93].

The effect of exercise training at high altitude has also been investigated in asthmatic populations. High-altitude treatment has long been recommended and applied as beneficial in asthma due to lower allergen exposure, less air pollution and lower air humidity [98,99]. Decreased air density at altitude also reduces airway resistance, which might increase exercise capacity [98]. A recent RCT found that a three-week comprehensive rehabilitation program with endurance training was highly effective in improving asthma control in patients with poorly controlled asthma—a benefit which was preserved after three months [83]. High-altitude training also led to better improvement in PEFR-variability, exercise capacity and airway inflammation as assessed by FeNO compared to low-altitude training [83].

Based on the available evidence, current guidelines recommend exercise as supplementary to medication therapy and support the inclusion of individuals with asthma in pulmonary rehabilitation programs [100,101] but there is insufficient evidence to date to recommend one form of exercise training over another [1]. Pre-exercise bronchodilators and gradual warm-up are indicated to prevent or minimize EIB [1,100].

To the extent of our knowledge, there has been no study to date to investigate the long-term effect of structured exercise training on PADL in asthmatic patients. This is an important knowledge gap that cannot be bridged by extrapolations from the acute effect of training on exercise capacity and other asthma-specific outcomes. PADL reflects complex behavioural patterns that may not be easily reformed by short-term or singular interventions. Paradigm from patients with COPD suggests that changing physical activity behaviour needs a long-lasting interdisciplinary approach, bringing together respiratory medicine, rehabilitation sciences, social sciences, and behavioural sciences, and best taken within the frame of pulmonary rehabilitation [102].

## 7. Additional Mechanisms of Benefit

Apart from psychological and the aforementioned physiological benefit through cardiopulmonary fitness and lung function, findings in human and animal studies suggest that the beneficial effects of exercise training on asthma are mediated by immune and metabolomic pathways.

The immune theory supports that physical activity reduces airway inflammation. It is based on the fact that airway inflammation is a principal feature of asthma shown to lead to increased risk of exacerbation and to increased asthma severity [103]; and that exercise training has proven systemic anti-inflammatory effects [104,105]. Potential mediating mechanisms include reductions in the serum T helper 2 proinflammatory cytokines interleukin (IL)-4, IL-5, and IL-6, IL-13–16, reductions in the monocyte chemoattractant protein 1 and keratinocyte chemoattractant protein (murine homologue to human IL-8), inhibition of nuclear factor kappa B (NFκB) activation, and increases in the anti-inflammatory cytokine IL-10, IL-1ra and circulating regulatory T cells. [84,106,107,108,109]. Increased PADL was also associated with lower systemic inflammation (for every increase of 1000 steps per day, high sensitivity-CRP was reduced by 17%) in patients with severe asthma after adjustment for confounders, albeit no associations were found with measures of eosinophilic airway inflammation [31]. Therefore, physical activity may potentially serve as a complementary therapy to target systemic inflammation in asthma [31].

At a tissue and organ level, aerobic exercise decreases eosinophilic airway inflammation, bronchial remodelling and respiratory mechanics in animal models of asthma as suggested by reductions in eosinophil count in bronchoalveolar lavage fluid, airway walls and sputum, decrease in FeNO as well as reductions in peribronchial inflammatory cell count, mucus synthesis, smooth muscle thickness and, airway resistance and elastance [108,109,110,111,112]. Exercise may also possibly improve the patency of bronchioles via epithelial stimulation and mucociliary clearance and improve smooth muscle function through deep inspiration and sigh rate [113]. Of note, some evidence suggests that exercise may not only reduce allergic airway inflammation as measured by FeNO, but also reduce nasal inflammation as measured by eosinophilic cell count and induce sustainable improvements in allergic symptoms [114].

Physical activity may also exert anti-inflammatory effects through metabolomic pathways such as increased production of butyrate, a circulating short chain fatty acid (SCFA), found in people with high cardiorespiratory fitness, independent of diet [115]. As discussed elsewhere [4], SCFAs have been shown to promote dendritic cell hematopoiesis and impair the promotion of Th2 effector cells, thus inhibiting allergic inflammation in animal models. Additionally, SCFAs decrease allergic effector T cell responses, promote formation of T regulatory cells and reduce NFκB activation in macrophages. Finally, exercise was shown to reverse the effects of a high-fat diet on gut microbiome in animal models [116,117,118].

## 8. Interventions Targeting Physical Activity in Asthma

### 8.1. Combined Exercise Training and Diet/Weight Loss Interventions

Obesity and asthma are associated diseases and obesity-related asthma is considered a distinct asthma phenotype [59]. Obesity reduces the responsiveness to inhaled corticosteroids [1] and increases the risk for developing asthma as well as the severity of asthma; epidemiological data show that obesity is more common among asthmatics than non-asthmatics, asthma is more common in obese than lean subjects and obesity is extremely common in patients with severe asthma [119,120]. Obesity has also been identified as a risk factor for insufficient physical activity in asthma [121] and both inactivity and obesity were shown to increase health care use in asthmatic patients [72].

Physical inactivity might explain the association between asthma and obesity as the linking element in establishment and sustainment of the vicious cycle of inactivity, obesity, and worse asthma control [9]. Of note, weight loss, whether by diet and exercise or by bariatric surgical intervention, reduces medication needs, improves lung function, asthma control, and asthma-related quality of life [1,122]. In addition, dietary habits have been associated with systemic inflammation, a condition associated with asthma as well as lung function and clinical outcomes, and epidemiological evidence suggests that a high fruit and vegetable diet is associated with lower risk of asthma and lung function decline [1]. There is also some evidence that low glycaemic index diet is associated with reductions in low-grade systemic inflammation (plasma IL-6 concentrations) and FEV_1_ [123], whereas low-fruit/low-vegetable diet, which is also low in antioxidants, led to increased circulating CRP and increased risk of asthma exacerbations [124].

The above line of evidence sets the scientific rationale for combined interventions targeting physical activity, diet, and weight management in the important subset of asthmatic patients with obesity. Accordingly, a RCT showed that a two-month high-intensity interval training (spinning), when combined with high protein/low glycemic index diet improved asthma control and asthma-related quality of life in nonobese adults with asthma [85], although these effects were proven non-sustainable at one-year follow-up [125]. Importantly, in a RCT by Freitas and colleagues, exercise training combined with a weight loss program improved PADL in 55 grade II obese adults with asthma compared to weight loss alone, suggesting that if combined, these intervention can encourage obese asthmatic patients to become physically active [59]. In this study, exercise training included supervised aerobic and resistance exercises twice weekly for three months plus recommendations for PADL and the weight loss program included 12 individual hypocaloric diet counselling sessions supported by behavioural techniques based on the transtheoretical model. After three months, patients who attended both the exercise training and weight loss programs presented a significant increase in daily step counts (3068 ± 2325 vs. 729 ± 1118 steps per day), time spent in MVPA (18.2 ± 17.9 vs. 7.9 ± 13.8 min per day), and the number of asthma symptom-free days (14.5 ± 9.6 vs. 8.6 ± 11.4 days/months) compared with patients who attended only the weight loss program, thus meeting satisfactorily current guidelines [59].

### 8.2. Pharmacological Interventions

At this time, evidence on the potential effect of available therapies on physical activity in asthma is scarce. In the eosinophilic phenotype of severe asthma, anti-IL-5 therapy enables reduction of asthma exacerbations and steroid use, better control of asthma and health-related quality of life, and improved lung function [126,127,128]. We have previously reported on the results of an ongoing study (NCT03739320), i.e., on the effects of anti-IL-5 therapy on PADL in patients with severe eosinophilic asthma [39]. At 12 months, mepolizumab therapy on top of existing, maximal, and optimised asthma treatment improved significantly daily moving time, step count, movement intensity, and weekly MET-min by 11% (*p* = 0.022), 14% (*p* = 0.022), 5% (*p* = 0.005) and 17% (*p* = 0.004), respectively compared to prior to biological therapy [39]. Of note, daily steps increased by > 600 (from 8930 ± 1887 to 9562 ± 2015), thus reaching the minimal important difference in COPD patients after pulmonary rehabilitation [129]. These preliminary results lend promise that anti-IL-5 therapy may be beneficial for PADL in patients with severe eosinophilic asthma, although a larger data sample is certainly required to adequately test this hypothesis. Nonetheless, the possibility that a single, add-on therapeutic intervention, may succeed in improving PADL in severe asthma is exciting and worth further consideration.

### 8.3. Behavioral Interventions

Increasing participation in PADL requires lifestyle interventions and conscious behaviour modifications [130]. Behaviour change interventions aim to develop participant’s confidence and self-efficacy to demonstrate the desired behaviour through motivation, education, action planning and support. Behaviour change techniques include individual goal setting and behavior contract, monitoring and feedback, avoidance strategies, and problem solving/coping planning [131]. Appropriate choice of the inhaler device based on patient characteristics, inhaler-technique training and counselling on the importance of adherence to therapy are also key elements for successful asthma management [132].

In a previous study examining the effect of education, positive affect, and self-affirmation on physical activity in patients with mild to moderate asthma, the authors concluded that a multiple-component protocol was effective at increasing energy expenditure, but an intervention to increase positive affect and self-affirmation was not effective within this protocol [133]. However, the inclusion of patients with only mild-to-moderate asthma, minimal impairment in physical fitness and limited comorbidities, and the quantification of physical activity through questionnaire limit the generalisability of these findings. A RCT in adults with moderate-to-severe asthma aiming to investigate the effects of a behaviour change intervention on increasing participation in physical activity and improving asthma clinical control is ongoing (NCT-03705702). The intervention is based on feedback and delivered over eight sessions as weekly one-on-one, face-to-face, 40-min consultations [131].

**Figure 1:**
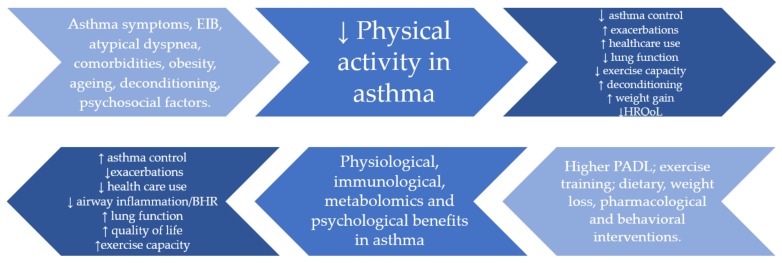
Conceptual model for the interrelationship between asthma, physical activity and targeted interventions. EIB: exercise-induced bronchoconstriction; PADL: physical activity in daily life; BHR: bronchial hyperresponsiveness.

A conceptual model for the interrelationship between asthma, physical activity, and targeted interventions is presented in Figure 1.

## 9. Conclusions

Our better understanding of the nature of asthma, the advent of effective pharmacological therapies, and relevant research have led to a paradigm shift in the management asthma with regards to physical activity over the course of the last decades. The ultimate aims of asthma management are required to include the minimization of symptoms so that individuals with asthma can lead normal lives, including engagement in recommended levels of PADL, exercise training or sports. Beyond its general health benefits, physical activity seems to be an important link in the successful management of asthma. Higher adherence to physical activity is associated with favourable clinical outcomes such as improved lung function, asthma control, exacerbation rate, and healthcare use. However, evidence shows that physical activity in asthma remains low, especially in the severe form. The relationship between asthma and physical activity is bidirectional and multifaceted and PADL represents a challenging area for interventions in that it reflects well-established behavioral patterns. Nonetheless, in the modern patient-centred era of healthcare service, reversing the negative loop to a virtuous cycle of enhanced physical activity and asthma control represents a meaningful and achievable goal. To this goal, well-designed studies examining the interrelationship between physical activity, airway inflammation, lung function, and clinical outcomes, as well as the effect of interventions promoting a physically active lifestyle in asthmatic individuals are urgently needed.
